# Experimental Study of the Reactions of Br Atoms with Thiirane and Nitrosyl Chloride

**DOI:** 10.3390/molecules30092058

**Published:** 2025-05-06

**Authors:** Yuri Bedjanian

**Affiliations:** Institut de Combustion, Aérothermique, Réactivité et Environnement (ICARE), CNRS, 45071 Orléans, France; yuri.bedjanian@cnrs-orleans.fr; Tel.: +33-238255474

**Keywords:** bromine atom, C_2_H_4_S, ClNO, kinetics, rate coefficient, temperature dependence

## Abstract

The kinetics of Br-atom reactions with C_2_H_4_S and ClNO were studied as a function of temperature at a total pressure of 2 Torr of helium using a discharge–flow system combined with mass spectrometry: Br + C_2_H_4_S → SBr + C_2_H_4_ (1) and Br + ClNO →BrCl + NO (2). The rate constant of reaction (1) was determined at T = 340–920 K by absolute measurements under pseudo-first-order conditions, either by monitoring the kinetics of Br-atom or C_2_H_4_S consumption in excess of C_2_H_4_S or of Br atoms, respectively, and by using a relative rate method: *k*_1_ = (6.6 ± 0.7) × 10^−11^ exp(−(2946 ± 60)/T) cm^3^molecule^−1^s^−1^ (where the uncertainties represent the precision at the 2σ level, the estimated total uncertainty on *k*_1_ being 15% at all temperatures). The rate coefficient of reaction (2), determined either from the kinetics of the formation of the reaction product, BrCl, or from the decays of Br-atoms in an excess of ClNO, showed non-Arrhenius behavior, being practically independent of temperature below 400 K and increasing significantly at temperatures above 500 K. The measured rate constant is well reproduced by a sum of two exponential functions: *k*_2_ = 1.2 × 10^−11^ exp(−19/T) + 8.0 × 10^−11^ exp(−1734/T) cm^3^ molecule^−1^ s^−1^ (with an estimated overall temperature-independent uncertainty of 15%) at T = 225–960 K.

## 1. Introduction

The peculiarity of the thiirane reactions with different atoms is that they proceed by desulfurization of C_2_H_4_S, resulting in almost stoichiometric formation of ethylene and corresponding sulfur-containing radicals. In this regard, these reactions, in addition to their theoretical interest, are of practical importance associated with laboratory studies of gas-phase kinetics, where they can potentially be used to generate various sulfur-containing radicals. Previous work has reported kinetic studies of the reactions of C_2_H_4_S with hydrogen [1,2,3], oxygen [4,5], sulfur [6,7,8] and chlorine atoms [9,10]. In this paper, we report the results of the first experimental study of the reaction of thiirane with bromine atoms:

Br + C_2_H_4_S → SBr + C_2_H_4_            Δ_r_*H*° = (9.18 ± 1.25) kJ mol^−1^ (1)

The enthalpies of formation used for the calculations of Δ_r_*H*° were the following: Δ_f_*H*(298K) = (111.87 ± 0.12), (82.3 ± 1.0), (52.45 ± 0.13) and 150.9 kJ mol^−1^ for Br [11], C_2_H_4_S [12], C_2_H_4_ [11] and SBr [13], respectively. In contrast to the previously studied reactions of thiirane with different atoms, reaction (1) is endothermic and is expected to proceed with a significant activation energy. The aim of this work was to measure the rate constant of reaction (1) as a function of temperature and to obtain information about the reaction mechanism by detecting the expected reaction product, the SBr radical.

In addition, as a part of this study, the rate constant of the reaction of bromine atoms with ClNO was measured. In fact, this reaction was used in the study of reaction (1) for the detection of Br atoms by mass spectrometry through their chemical conversion to the stable species BrCl and as a reference reaction in the relative measurements of *k*_1_:

Br + ClNO → BrCl + NO(2)

So far, the reaction rate constant has only been measured in two studies, by Clyne and Cruse [14] at room temperature and by Abbatt et al. [15] in the temperature range (229–424) K. In the present work, we report the measurements of the rate constant in an extended temperature range, T = 225–960 K.

## 2. Results and Discussion

### 2.1. Rate Constant of Reaction (1)

#### 2.1.1. Absolute Measurements of *k*_1_: Kinetics of Br Atom Consumption

**Figure 1:**
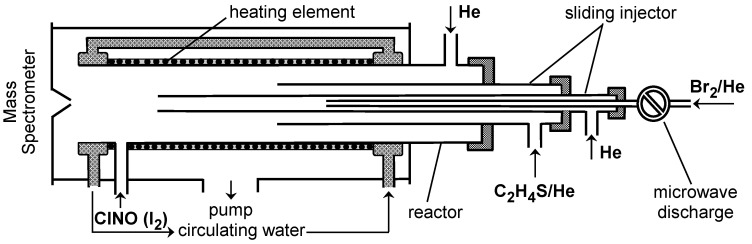
High-temperature flow reactor: configuration used in absolute measurements of the rate constant of reaction (1).

Br atoms were generated from the microwave discharge in trace amounts of molecular bromine in He (Figure 1). The dissociation of Br_2_ was verified by mass spectrometry to be between 50 and 95% (increasing with decreasing Br_2_ concentration).

Two chemical conversion methods were used to detect Br atoms. In the first, Br atoms were converted to BrCl by reaction (2) with excess ClNO ([ClNO] = (1–2) × 10^14^ molecule cm^−3^) prior to introduction into the mass spectrometer (Figure 1) and detected at m/z = 114/116 (BrCl^+^):

*k*_2_ = 1.2 × 10^−11^ exp(−19/T) + 8.0 × 10^−11^ exp(−1734/T) cm^3^ molecule^−1^ s^−1^ (T = 225–960 K, this work). The second method used the reaction of Br atoms with excess I_2_ ([I_2_] = (2–3) × 10^13^ molecule cm^−3^), converting the Br atoms to IBr, followed by mass spectrometric detection of the IBr^+^ ion at m/z = 206/208:

Br + I_2_ → IBr + Br (3)

*k*_3_ = (1.20 ± 0.15) × 10^−10^ cm^3^ molecule^−1^ s^−1^ (T = 298 K) [16]. To our knowledge, only the room temperature value of *k*_3_ is available in the literature. However, the rate constant of reaction 3 is not expected to be strongly temperature dependent, given the very high value of *k*_3_ at T = 298 K.

The rate constant of reaction (1) was determined under pseudo-first-order conditions from the kinetics of Br-atom ([Br]_0_ = (1.0–2.5) × 10^11^ molecule cm^−3^) consumption monitored under an excess concentration of C_2_H_4_S (Table 1).

**Figure 2:**
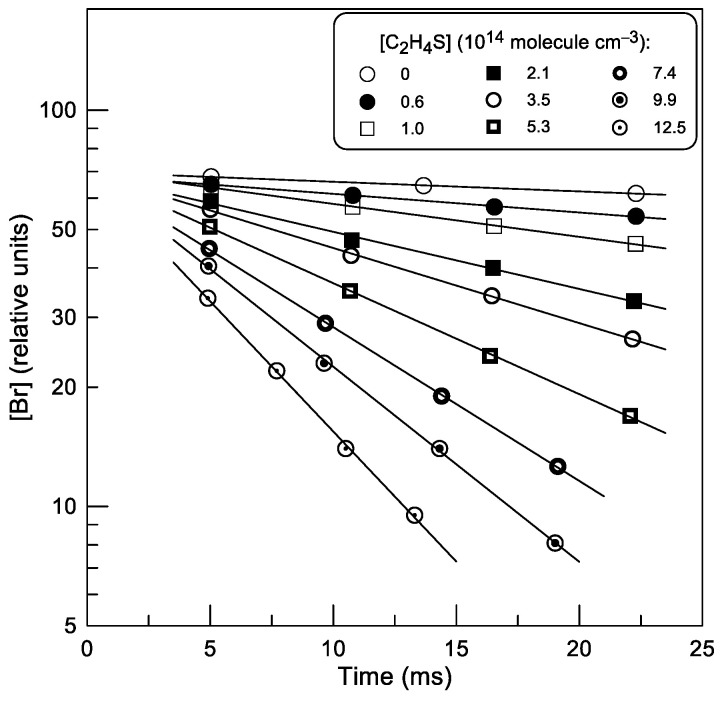
Reaction (1): plots of Br-atom concentration against reaction time for various concentrations of C_2_H_4_S (T = 470 K).

Consumption of the excess reactant, C_2_H_4_S, was negligible due to its high excess over Br atoms. Figure 2 shows examples of exponential decays of Br atoms in the presence of different concentrations of thiirane in the reactor: [Br] = [Br]_0_ × exp(−*k*_1_′ × t), where *k*_1_′ = *k*_1_ × [C_2_H_4_S] + *k*_w_ is a pseudo-first order rate constant and *k*_w_ is the rate of heterogeneous loss of Br atoms.

**Figure 3:**
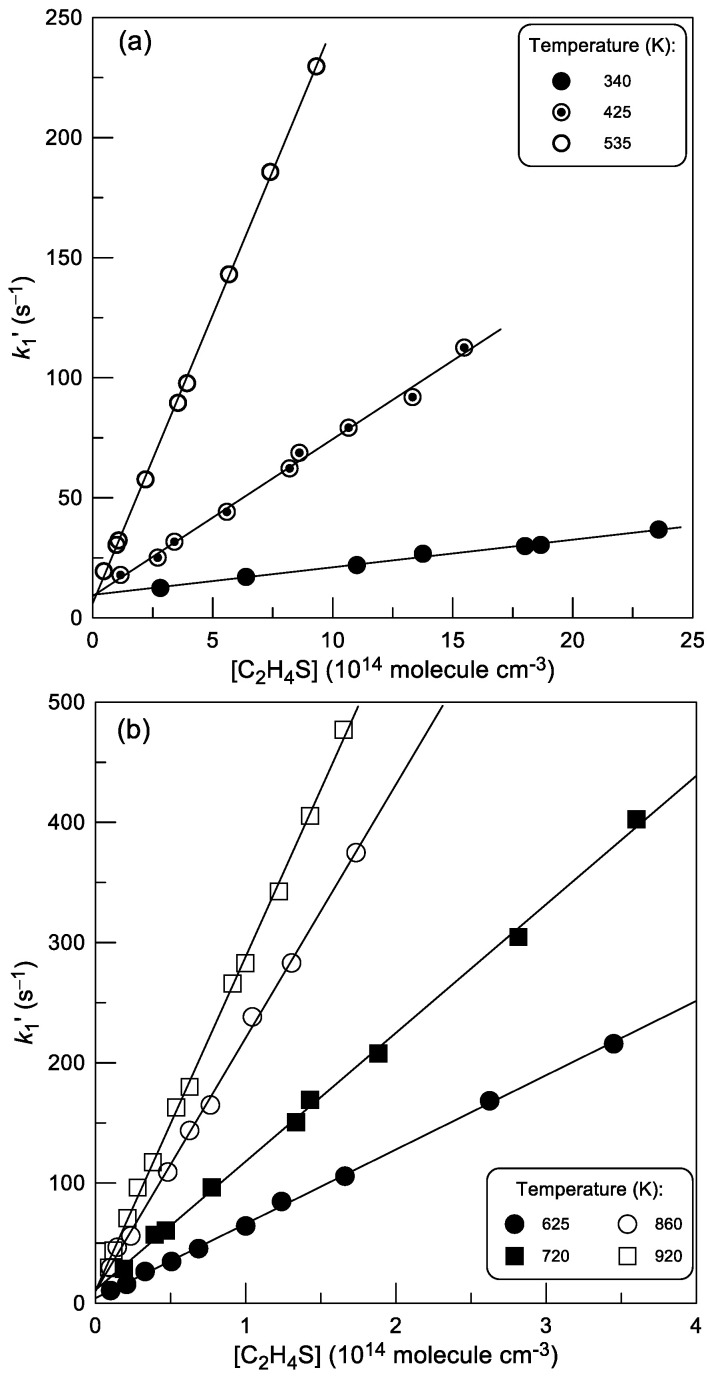
Reaction (1): pseudo-first-order rate constant *k*_1_′ = *k*_1_ × [C_2_H_4_S] + *k*_w_ as a function of thiirane concentration at different temperatures: (**a**) T = 340, 425 and 535 K; (**b**) T = 625, 720, 860 and 920 K.

Examples of second-order plots observed at different temperatures are shown in Figure 3. All data in Figure 3 were measured with Br detected as BrCl after its chemical conversion in reaction (2).

**Figure 4:**
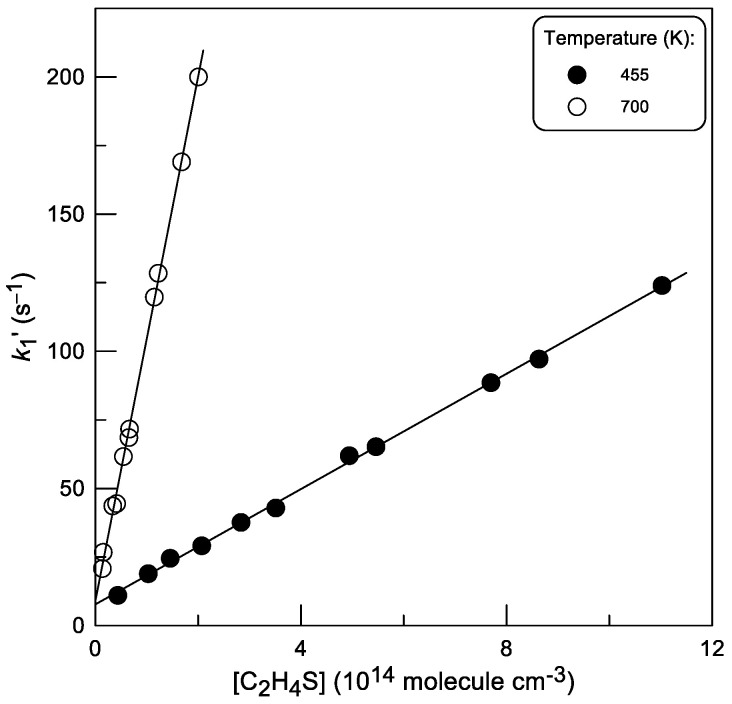
Pseudo-first-order rate constant *k*_1_′ as a function of thiirane concentration measured with Br detection via conversion to IBr.

Similar quality data were obtained when Br was detected by conversion to IBr in reaction (3) (Figure 4). All pseudo-first order rate constants, *k*_1_′, were corrected for axial and radial diffusion of Br atoms [17]: *k*_1_′(corrected) = *k*_1_′ × (1 + *D*_eff_*k*_1_′/*v*^2^), where *D*_eff_ = *D* + *r*^2^*v*^2^/48*D* (*D* is the diffusion coefficient of Br in He calculated as *D*_0_ = 480 × (T/298)^1.85^ Torr cm^−2^ s^−1^ [18], *v* is a linear flow velocity and *r* is the reactor radius). The corrections were typically a few percent.

The rate constants of the reaction (1), obtained from a linear least-squares fit of the *k*_1_′ data as a function of [C_2_H_4_S], are given in Table 1. Good agreement can be observed between the results obtained with two methods of Br-atom detection, using chemical conversion of Br to BrCl and IBr in reactions (2) and (3), respectively.

SBr was directly detected by mass spectrometry as the product of reaction (1) at m/z = 111/113 (SBr^+^). The addition of I_2_ at the end of the reactor (Figure 1) resulted in the appearance of mass spectrometric signals at m/z = 238/240 (ISBr^+^) and m/z = 159 (IS^+^) (ISBr fragment), indicating the occurrence of the reaction SBr + I_2_ → ISBr + I, leading to the formation of ISBr. Although the absolute concentrations of SBr were not measured, judging from the relative mass spectrometric signals, the SBr formation channel is the main and, probably, the only reaction channel under the experimental conditions of the study. Attempts to detect HBr as another potential reaction product showed that its yield was less than 3% at T = 920 K, the highest temperature of the study.

The thermal decomposition of thiirane under the experimental conditions of the study was rather slow, at least without any noticeable effect on the C_2_H_4_S concentration along the reaction zone. An indirect indicator of the C_2_H_4_S decomposition was the formation of S_2_ (detected at m/z = 64 (S_2_^+^), ~0.3% of the C_2_H_4_S signal intensity in experiments at T = 920 K), most likely, in the reaction sequence:

C_2_H_4_S + M → C_2_H_4_ + S + M (4)

S + C_2_H_4_S → S_2_ + C_2_H_4_ (5)

Secondary reactions leading to additional consumption or reproduction of Br atoms in the chemical system used with potential impact on *k*_1_ measurements can be discussed. First, the influence of the secondary reaction of Br atoms with C_2_H_4_ formed in reaction (1) can be neglected due to the low initial concentrations of Br atoms used in the kinetic measurements (and hence the low concentration of the reaction product C_2_H_4_). For the same reason, the potential consumption of Br in the reaction with SBr and Br reproduction in the reverse reaction of SBr with C_2_H_4_ should not have a significant effect on the *k*_1_ measurements. Other potential candidates for reproducing Br atoms include the reactions of SBr:

SBr + Br_2_ → Br + SBr_2_ (6)

SBr + SBr → 2Br + S_2_ (7)

SBr + SBr → Br + SSBr(8)

To the best of our knowledge, there is a paucity of data in the literature on the rate constants of these reactions. However, their significant influence on the present measurements can be ruled out based on general considerations. Thus, a significant effect of reaction (6) on Br kinetics under experimental conditions is unlikely given very low Br_2_ concentrations (≤3 × 10^10^ molecule cm^−3^) in the reactor. Reaction (7) is endothermic by 50.5 kJ mol^−1^ [11,13]. There are no thermochemical arguments (the enthalpy of SSBr formation is unknown) to exclude the occurrence of reaction (8) in our experiments. However, even if reaction (8) occurs, its impact should be limited given the low initial concentrations of Br atoms (and hence SBr) used in the kinetic measurements.

#### 2.1.2. Absolute Measurements of *k*_1_: Kinetics of C_2_H_4_S Consumption

**Figure 5:**
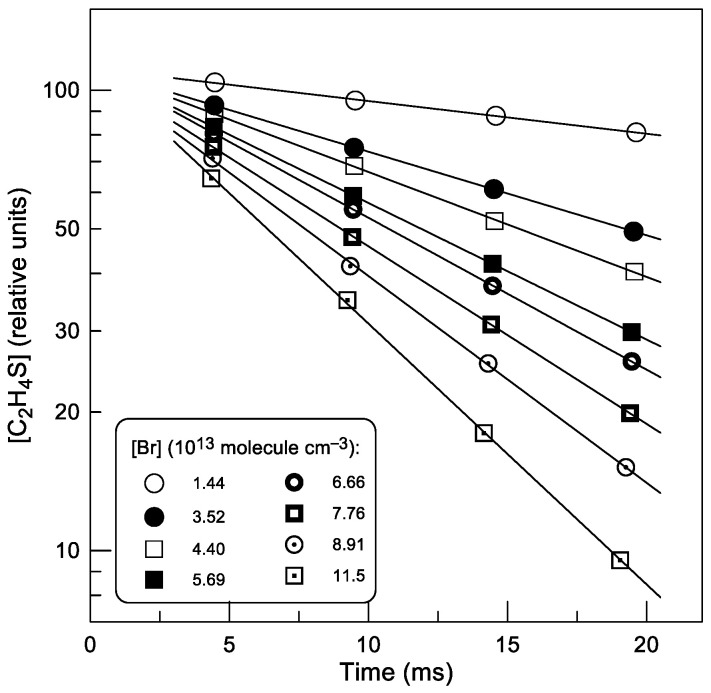
Reaction (1): kinetics of C_2_H_4_S consumption observed at different concentrations of Br atoms in the reactor (T = 735 K).

Although potential secondary chemistry appears to have a limited impact on the measurements of *k*_1_, to be more confident of its minor role we performed experiments in which *k*_1_ was determined from C_2_H_4_S decays ([C_2_H_4_S]_0_ = (2–5) × 10^11^ molecule cm^−3^) monitored in excess of Br atoms (Table 1). Examples of the concentration vs. time profiles of thiirane observed at T = 735 K with different concentrations of Br atoms are shown in Figure 5: [C_2_H_4_S] = [C_2_H_4_S]_0_ × exp(−*k*_1_′ × t), where *k*_1_′ = *k*_1_ × [Br] is the pseudo-first order rate constant and [Br] corresponds to the average Br-atom concentration along the reaction zone. In fact, the Br-atom consumption was insignificant and was mainly due to the heterogeneous loss of the atoms (*k*_w_ < 10 s^−1^). The Br atoms consumed in the reaction with C_2_H_4_S were, at least, partially reproduced in reaction (6) between SBr, the product of reaction (1), and Br_2_ still present in the reactor as a precursor of the Br atoms (incomplete dissociation in the microwave discharge).

**Figure 6:**
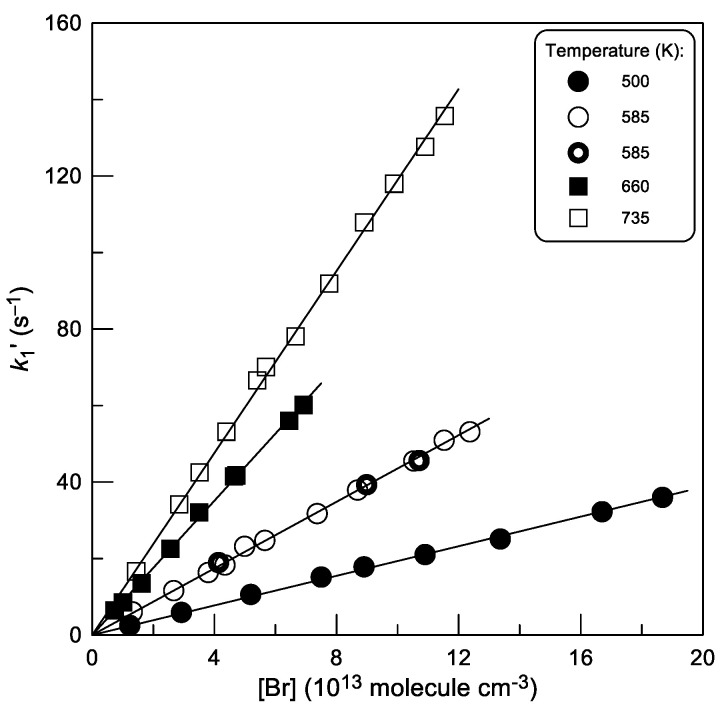
Reaction (1): pseudo-first-order rate constant *k*_1_′ = *k*_1_ × [Br] as a function of Br-atom concentration at different temperatures. Different symbols at T = 585 K correspond to experiments with different initial concentrations of C_2_H_4_S (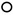: 2.0 × 10^11^; 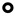: 5.0 × 10^11^ molecule cm^−3^).

The second-order plots observed in this series of experiments at different temperatures are shown in Figure 6. The corresponding values of *k*_1_ derived from linear least squares fit of these data at each temperature are given in Table 1.

#### 2.1.3. Relative Measurements of *k*_1_

In addition to the absolute measurements, at two temperatures, the rate constant of reaction (1) was measured relative to that of the reaction of Br atoms with ClNO (reaction (2)). The experiments consisted of measuring the yield of BrCl as a function of the [C_2_H_4_S]/[ClNO] ratio after complete consumption of [Br]_0_ (≈3 × 10^11^ molecule cm^−3^) in the reaction with a mixture of C_2_H_4_S and ClNO ([C_2_H_4_S] = (0.16–3.37) × 10^14^ and (0.15–5.41) × 10^14^ molecule cm^−3^, ([ClNO] ≈ 1 × 10^13^ and 2.3 × 10^13^ molecule cm^−3^ at T = 720 and 860 K, respectively):

(9) $$\left[\mathrm{BrCl}\right]=\frac{k_{2}[\mathrm{ClNO}]}{k_{2}\left[\mathrm{ClNO}\right]+k_{1}\left[C_{2}H_{4}S\right]+k_{w}}\times {\left[\mathrm{Br}\right]}_{0}$$

Transforming this expression, we obtain:

(10) $$\frac{{\left[\mathrm{Br}\right]}_{0}}{\left[BrCl\right]}-1=\frac{k_{1}[C_{2}H_{4}S]}{k_{2}\left[ClNO\right]}+\frac{k_{w}}{k_{2}[ClNO]}$$

**Figure 7:**
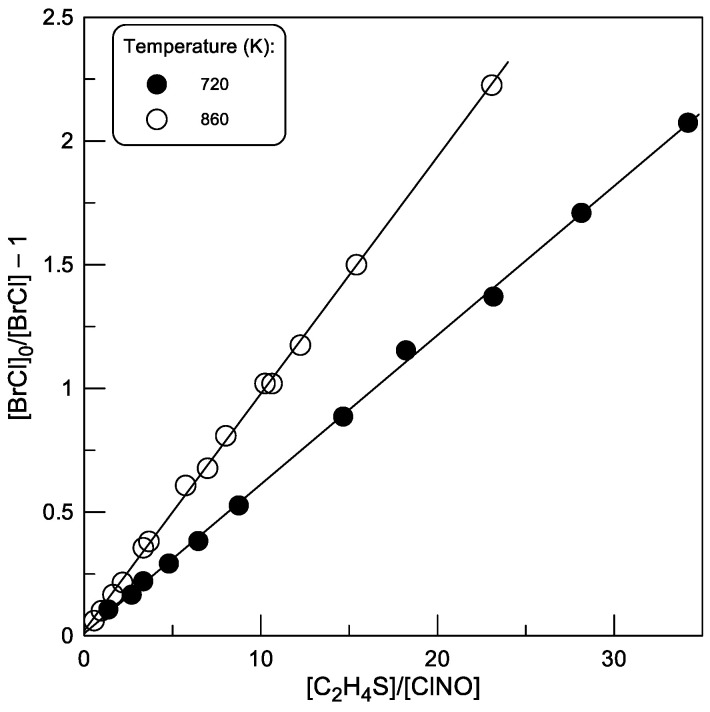
Reaction (1): yield of BrCl upon titration of Br atoms in reaction with C_2_H_4_S/ClNO mixtures at T = 720 and 860 K.

At a constant concentration of ClNO, the second term in Equation (10) is constant, and the *k*_1_/*k*_2_ ratio can be determined as the slope of the linear dependence of ([Br]_0_/[BrCl] − 1) on the [C_2_H_4_S]/[ClNO] ratio. The initial concentration of Br atoms, [Br]_0_, could be expressed in terms of the BrCl signal by titration of Br in reaction with ClNO in the absence of C_2_H_4_S in the reactor ([Br]_0_ = [BrCl]_0_), thus avoiding the measurement of absolute Br and BrCl concentrations. The observed experimental data are shown in Figure 7. The final values of *k*_1_, calculated using the ratios of *k*_1_ to *k*_2_ determined from the slopes of the straight lines in Figure 7 and *k*_2_ measured in the present work (see below), are given in Table 1.

#### 2.1.4. Temperature Dependence of *k*_1_

**Figure 8:**
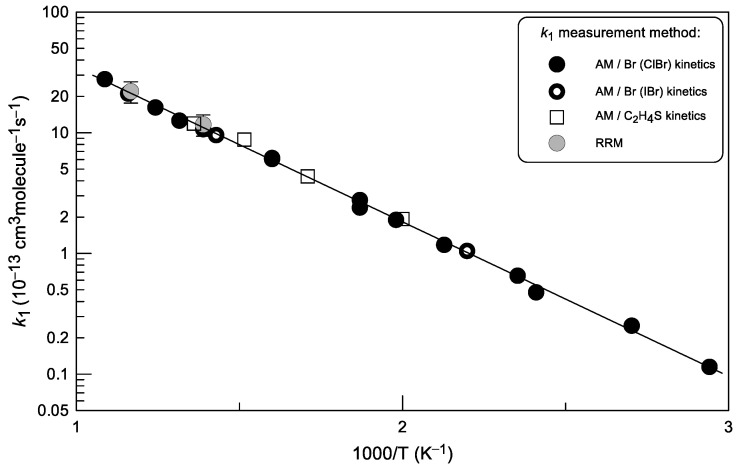
Summary of the measurements of the rate constant of reaction (1). Error bars shown for the relative measurements (RRM) of *k*_1_ correspond 20% uncertainty; the uncertainty of 15% estimated for absolute measurements (AM) of *k*_1_ corresponds to the size of the corresponding symbols.

The results of all measurements of the rate constant of the reaction (1) are displayed in Figure 8.

A very good agreement can be observed between the values of *k*_1_ measured with the relative and two absolute measurement methods. The combined uncertainty on the absolute measurements of *k*_1_ was estimated to be ≤15% by adding in quadrature statistical error (≤3%) and those on the measurements of the absolute concentration of C_2_H_4_S (Br) (~10%), flows (3%), pressure (2%) and temperature (1%).

The observed temperature dependence of *k*_1_ can be described by the following Arrhenius expression:

*k*_1_ = (6.6 ± 0.7) × 10^−11^ exp(−(2946 ± 60)/T) cm^3^molecule^−1^s^−1^

in the temperature range from 340 to 920 K and with 2σ uncertainties representing the precision of the fit. It can be noted that the experimentally determined activation energy (≈24.5 kJ mol^−1^) significantly exceeds the endothermicity of the reaction (≈9.2 kJ mol^−1^), which obviously indicates that reaction (1) proceeds through a transition state located well above the energy of the reaction products. Consequently, the reverse addition–elimination reaction of SBr radicals with C_2_H_4_ is expected to proceed with a significant activation energy.

### 2.2. Rate Constant of Reaction (2)

#### 2.2.1. Kinetics of Br Atom Consumption

**Figure 9:**
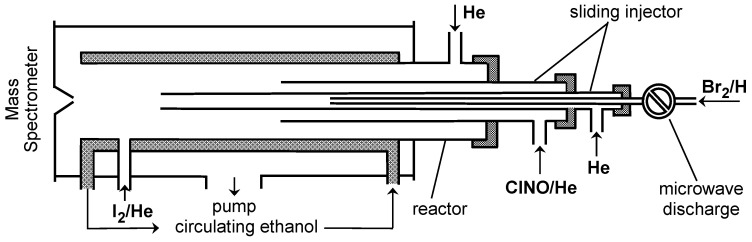
Diagram of the low temperature flow reactor: configuration used in the absolute measurements of *k*_2_.

In this series of experiments, the rate constant of reaction (2) was determined under pseudo-first order conditions by monitoring the kinetics of Br-atom consumption in excess of ClNO. Br atoms were converted to IBr prior to sampling in the mass spectrometer and were detected at m/z = 206/208 (Figure 9). As expected, exponential [Br] decays were observed, [Br] = [Br]_0_ × exp(−*k*_2_′ × t), where [Br] and [Br]_0_ ([Br]_0_ = (1.0–2.5) × 10^11^ molecule cm^−3^) are the time-dependent and initial concentrations of Br atoms, respectively, and *k*_2_′ = *k*_2_ × [ClNO] + *k*_w_. The pseudo-first-order rate constants were determined from an exponential fit to the Br atom consumption kinetics. Diffusion corrections applied to *k*_2_′ were <10%. The consumption of excess reactant, ClNO, in reaction (2), although insignificant (<10%), was taken into account. At the highest temperature of the study, T = 960 K, a slow thermal decomposition of ClNO (within a few %) was observed, as evidenced by the formation of Cl_2_ as a result of two consecutive reactions:

ClNO + M → Cl + NO + M(11)

Cl + ClNO → Cl_2_ + NO (12)

This process did not affect the Br-atom kinetics; appropriate corrections were made to the ClNO concentration.

**Figure 10:**
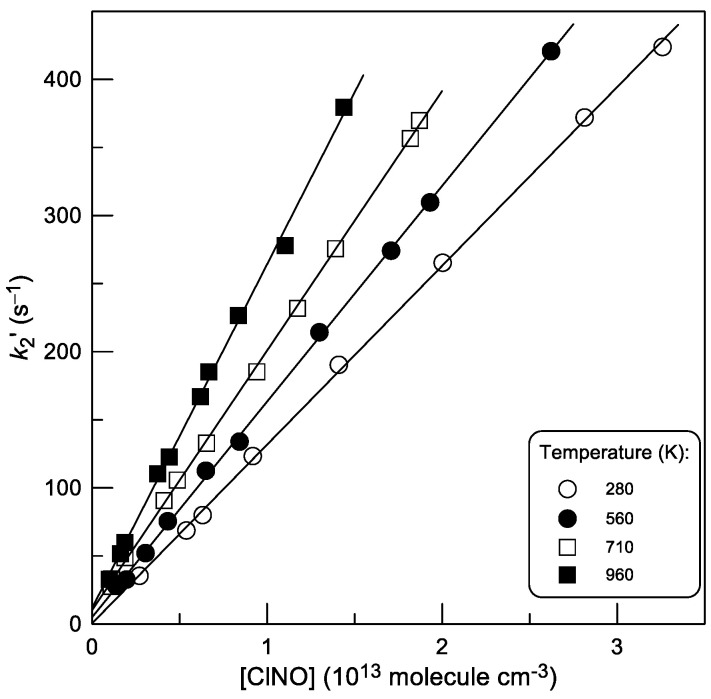
Reaction (2): pseudo-first-order rate constant *k*_2_′ = *k*_2_ × [ClNO] + *k*_w_ as a function of ClNO concentration at different temperatures.

Examples of the dependence of the pseudo-first order rate constant, *k*_2_′, on the concentration of ClNO at different temperatures are shown in Figure 10. The values of *k*_2_ at different temperatures, obtained by linear least squares fitting of kinetic data similar to those shown in Figure 10, are given in Table 2.

#### 2.2.2. Kinetics of BrCl Production

**Figure 11:**
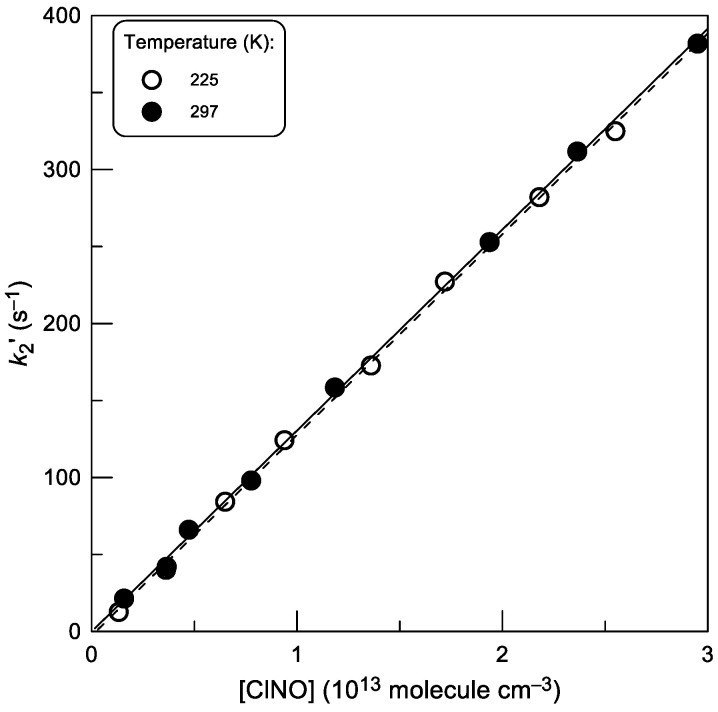
Reaction (2): pseudo-first-order rate constant *k*_2_′ = *k*_2_ × [ClNO] as a function of ClNO concentration at T = 225 and 297 K.

At low temperatures, the detection of Br atoms by their conversion to IBr was difficult due to I_2_ condensation. For this reason, the reaction rate constant was determined from the kinetics of the formation of the reaction product, BrCl, monitored in an I_2_-free system under experimental conditions similar to those used above in the study of Br kinetics. The kinetics of the reaction product can be approximated by the following expression: [BrCl] = [Br]_0_ − [Br]_0_ × exp(−*k*_2_′t) where *k*_2_′ ≈ *k*_2_ × [ClNO], provided that *k*_w_ << *k*_2_ × [ClNO]. The latter condition is satisfied at least at high ClNO concentrations, given that *k*_w_ < 10 s^−1^. A rearrangement of this expression gives [BrCl]_∞_ − [BrCl] = [BrCl]_∞_ × exp(−*k*_2_′t), where [BrCl]_∞_ = [Br]_0_ was determined by titration of bromine atoms with high concentrations of ClNO. In experiments, the kinetics of [BrCl] growth was monitored and [BrCl]_∞_ − [BrCl] was plotted as a function of time, yielding the values of *k*_2_′. Examples of the dependence of the pseudo-first order rate constant, *k*_2_′ ≈ *k*_2_ × [ClNO], on ClNO concentration are shown in Figure 11. The slopes of the straight lines in Figure 11 provide the values of *k*_2_ at corresponding temperatures. All *k*_2_ data determined within this experimental approach are shown in Table 2.

#### 2.2.3. Temperature Dependence of *k*_2_

**Figure 12:**
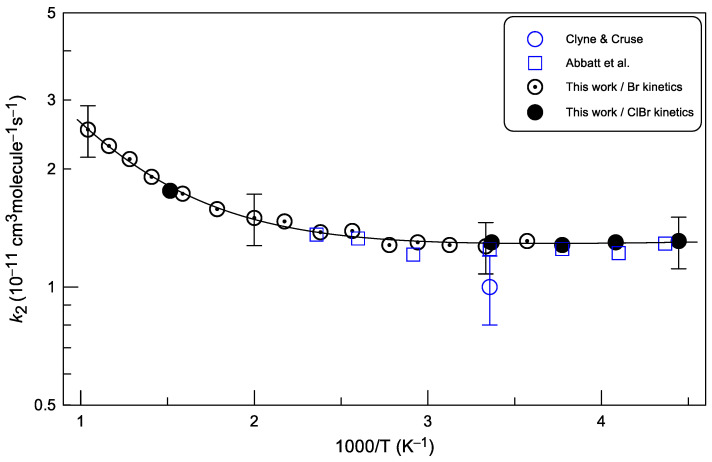
Summary of the measurements of the rate constant of reaction (2). Partially shown error bars on the present data correspond to the uncertainty of 15% estimated for *k*_1_ measurements. Previous work: Clyne and Cruse [14], Abbatt et al. [15].

The results of the previous and current measurements of *k*_2_ are shown in Figure 12.

Abbatt et al. [15] measured the rate constant of reaction (2) as a function of temperature (T = 225–425 K) in a discharge flow reactor using resonance fluorescence technique to detect Br atoms. The results obtained by these authors for *k*_2_ are in excellent agreement with the present measurements (Figure 12). The room temperature value of *k*_2_ = (1.0 ± 0.2) × 10^−11^ cm^3^ molecule^−1^s^−1^ reported by Clyne and Cruse [14] is somewhat lower, but agrees with the present data within the reported uncertainties.

The present measurements, carried out over an extended temperature range, revealed a curved temperature dependence of the rate constant: *k*_2_, which is practically independent of temperature below 400 K, increases significantly at temperatures above 500 K. The solid line in Figure 12 represents a fit of the present experimental *k*_2_-values to the sum of two exponential functions:

*k*_2_ = 1.2 × 10^−11^ exp(−19/T) + 8.0 × 10^−11^ exp(−1734/T) cm^3^ molecule^−1^ s^−1^

This expression reproduces all experimental data to within 3% and is recommended from the present work for the rate constant of reaction (2) in the temperature range T = (225–960) K with conservative independent of temperature uncertainty of 15%. The experimental data can also be described with temperature independent *k*_2_ = (1.3 ± 0.2) × 10^−11^ cm^3^ molecule^−1^ s^−1^ at T = (225–340) K and modified Arrhenius expression of *k*_2_ = 6.33 × 10^−16^ × (T)^1.47^exp(455/T) cm^3^ molecule^−1^ s^−1^ (uncertainty of 15%) at T = 340–960 K.

In the previous work [19], a non-Arrhenius curved temperature dependence was observed for the rate constant of the ClNO reaction with OH radicals. The reaction was shown to proceed via two competing pathways with comparable branching ratios: Cl-atom abstraction,

OH + ClNO → HOCl + NO(13)

and the exchange reaction (14), which probably proceeds by an addition–elimination mechanism,

OH + ClNO → Cl + HONO (14)

Interestingly, a non-Arrhenius behavior (increasing upward curvature of the reaction rate constant with temperature) was observed for both reaction channels.

For the reaction of Br atoms with ClNO, the second possible channel is too endothermic [11] to compete with the BrCl forming pathway under the experimental conditions of the study:

Br + ClNO → Cl + BrNO         Δ_r_*H*° = 37.3 kJ mol^−1^ (15)

At this stage, it is difficult to clearly explain the observed curvature of the temperature dependence of *k*_2_. One possible reason is the complex nature of reaction (2), including direct Cl-atom abstraction by the Br-atom and the Br addition–BrCl elimination mechanism.

## 3. Materials and Methods

All measurements were performed at a total helium pressure of approximately 2 Torr using a discharge flow reactor and a modulated molecular beam mass spectrometer with electron impact ionization operating at 30 eV energy.^1,4^ The reaction time was determined by the position of the tip of the movable injector relative to the sampling cone of the mass spectrometer (Figure 1 and Figure 9) and by the linear flow velocity in the reactor ((750–2520) and (1780–2650) cm s^−1^ for the study of reactions (1) and (2), respectively). The chemical composition of the reactive system in the reactor was monitored by sampling the gas-phase molecules from the flow reactor and detecting them with the mass spectrometer. Two flow reactors were used to provide an extended temperature range for kinetic measurements. The high-temperature reactor consisted of an electrically heated quartz tube (45 cm long and 2.5 cm i.d.) with water-cooled attachments (Figure 1) [20]. Water cooling was necessary to prevent overheating of the mass spectrometer sampling cone (Pyrex) and the main reactor vacuum connections. The temperature in the reactor was measured with a K-type thermocouple positioned in the middle of the reactor in contact with its outer surface [20]. The second flow reactor used at low temperatures consisted of a Pyrex tube (45 cm long and 2.4 cm i.d., coated with halocarbon wax) with a jacket for the circulation of a thermostated liquid (ethanol) (Figure 9).

Gas mixtures were prepared in 10 L glass cylinders by adding helium to a known amount (pressure) of compounds up to a total pressure of 1 atmosphere. Absolute concentrations of Br atoms, generated in a microwave discharge of Br_2_/He mixtures, were determined by measuring the dissociated fraction of Br_2_ (Δ[Br_2_]) and/or the concentration of ClNO consumed (Δ[ClNO]) upon titration of Br atoms in the reaction (2): [Br] = [BrCl] = 2Δ[Br_2_] = Δ[ClNO]. The results of the two calibration methods (by Br_2_ and ClNO) were in agreement within 5%. The absolute concentrations of C_2_H_4_S, ClNO and Br_2_ in the reactor were calculated from their flow rates obtained from pressure drop measurements in their mixtures in He (≈5%, 20% and 10%, respectively) stored in calibrated volumes.

The purities of the gases used were as follows: He > 99.999% (Alphagaz, Air Liquide France Industrie, Paris, France); Br_2_ > 99.99% (Aldrich, St. Louis, MO, USA); F_2_, 5% in helium (Alphagaz); C_2_H_4_S (Merck, Merck SA, Lyon, France), 98%. ClNO was synthesized in the laboratory using a mixture of Cl_2_ and NO [21]. The content of Cl_2_ and NO impurities in ClNO was estimated to be less than 1 and 2%, respectively.

## Figures and Tables

**Table 1:** Summary of the measurements of the rate constant of reaction (1).

*T* (K)	[Excess Reactant] *^a^*	*k*_1_ *^b^*	Method *^c^*
340	2.82–23.6	0.115	AM/Br (ClBr) kinetics
370	0.48–8.31	0.252	AM/Br (ClBr) kinetics
415	0.70–12.4	0.476	AM/Br (ClBr) kinetics
425	1.16–15.5	0.654	AM/Br (ClBr) kinetics
455	0.44–11.0	1.05	AM/Br (IBr) kinetics
470	0.57–12.5	1.18	AM/Br (ClBr) kinetics
500	0.12–1.87	1.93	AM/C_2_H_4_S kinetics
505	0.79–6.50	1.90	AM/Br (ClBr) kinetics
535	0.40–6.02	2.77	AM/Br (ClBr) kinetics
535	0.46–9.32	2.40	AM/Br (ClBr) kinetics
585	0.13–1.24	4.35	AM/C_2_H_4_S kinetics
625	0.10–3.45	6.18	AM/Br (ClBr) kinetics
625	0.34–6.32	6.06	AM/Br (ClBr) kinetics
660	0.07–0.69	8.77	AM/C_2_H_4_S kinetics
700	0.14–2.01	9.56	AM/Br (IBr) kinetics
720	0.19–3.60	10.7	AM/Br (ClBr) kinetics
720		11.7	RRM
735	0.14–1.15	11.9	AM/C_2_H_4_S kinetics
760	0.14–1.40	12.6	AM/Br (ClBr) kinetics
805	0.07–1.29	16.2	AM/Br (ClBr) kinetics
857		22.0	RRM
863	0.10–1.73	21.1	AM/Br (ClBr) kinetics
920	0.09–1.65	27.8	AM/Br (ClBr) kinetics

*^a^* units of 10^14^ molecule cm^−3^. *^b^* units of 10^−13^ cm^3^molecule^−1^s^−1^; statistical 2σ uncertainty is (2–3)%, total estimated uncertainty on *k*_1_ is 15% and 20% for absolute and relative measurements, respectively. *^c^* AM/Br (ClBr) and AM/Br (IBr): absolute measurements of *k*_1_ from Br kinetics in an excess of C_2_H_4_S with Br detection as ClBr and IBr (see text), respectively; AM/C_2_H_4_S kinetics: absolute measurements of *k*_1_ from kinetics of C_2_H_4_S in an excess of Br atom; RRM: relative rate method.

**Table 2:** Summary of the measurements of the rate constant of reaction (2).

*T* (K)	[ClNO] *^a^*	*k*_1_ *^b^*	Reactor Surface *^c^*	Method *^d^*
225	0.12–2.52	1.31	HW	BrCl kinetics
245	0.16–2.83	1.30	HW	BrCl kinetics
265	0.27–2.68	1.28	HW	BrCl kinetics
280	0.27–3.26	1.31	HW	Br kinetics
297	0.16–2.95	1.30	HW	BrCl kinetics
300	0.24–3.00	1.27	Q	Br kinetics
320	0.17–3.23	1.28	HW	Br kinetics
340	0.19–3.39	1.30	Q	Br kinetics
360	0.10–4.08	1.28	Q	Br kinetics
390	0.30–2.67	1.39	Q	Br kinetics
420	0.14–3.69	1.38	Q	Br kinetics
460	0.22–2.44	1.47	Q	Br kinetics
500	0.34–6.32	1.50	Q	Br kinetics
560	0.14–2.62	1.58	Q	Br kinetics
630	0.14–2.01	1.73	Q	Br kinetics
660	0.16–1.24	1.76	Q	BrCl kinetics
710	0.11–1.87	1.91	Q	Br kinetics
780	0.09–1.41	2.12	Q	Br kinetics
860	0.10–1.35	2.29	Q	Br kinetics
960	0.10–1.44	2.52	Q	Br kinetics

*^a^* Units of 10^13^ molecule cm^−3^. *^b^* units of 10^−11^ cm^3^molecule^−1^s^−1^; statistical 2σ uncertainty is (2–3)%, total estimated uncertainty on *k*_1_ is 15%. *^c^* HW: halocarbon wax; Q: quartz. *^d^* Br kinetics: *k*_1_ determined from kinetics of Br consumption; BrCl kinetics: *k*_1_ determined from kinetics of BrCl production.

## Data Availability

The data supporting reported results are available in this article.
